# Cytokines and the Pathogenesis of Macular Edema in Branch Retinal Vein Occlusion

**DOI:** 10.1155/2019/5185128

**Published:** 2019-05-02

**Authors:** Hidetaka Noma, Kanako Yasuda, Masahiko Shimura

**Affiliations:** Department of Ophthalmology, Hachioji Medical Center, Tokyo Medical University, Tokyo, Japan

## Abstract

Branch retinal vein occlusion (BRVO) is a very common retinal vascular problem in patients with lifestyle-related diseases, such as hypertension and arteriosclerosis. In patients with BRVO, development of macular edema is the main cause of visual impairment. BRVO is still a controversial condition in many respects. Over the years, various methods such as laser photocoagulation have been tried to treat macular edema associated with BRVO, but the results were not satisfactory. After vascular endothelial growth factor (VEGF) was found to have an important role in the pathogenesis of macular edema in BRVO patients, treatment of this condition was revolutionized by development of anti-VEGF therapy. Although macular edema improves dramatically following intraocular injection of anti-VEGF agents, repeated recurrence and resistance of edema is a major problem in some BRVO patients. This suggests that factors or cytokines other than VEGF may be associated with inflammation and retinal hypoxia in BRVO and that the pathogenesis of macular edema is complicated. The present review assesses the role of various factors and cytokines in the pathogenesis of macular edema associated with BRVO. We present a mechanism that is not only plausible but should also be useful for developing new therapeutic strategies.

## 1. Introduction

Branch retinal vein occlusion (BRVO) is a very common retinal vascular condition in patients who have lifestyle-related diseases, including hypertension and arteriosclerosis. Because macular edema is the main cause of visual impairment in BRVO, various approaches have been tried to treat it, including laser photocoagulation, but satisfactory outcomes were not achieved [1]. However, management of macular edema was revolutionized by the development of treatment targeting vascular endothelial growth factor (VEGF) [2]. Macular edema improves dramatically after injection of anti-VEGF agents, suggesting that VEGF plays a central role in this condition associated with BRVO. However, macular edema recurs in some patients or is resistant to anti-VEGF therapy, indicating the involvement of other agents. This article reviews the pathogenesis of macular edema in BRVO from the perspective of the role of VEGF and various other factors/cytokines.

## 2. Pathogenesis of Macular Edema in BRVO

**Figure 1:**
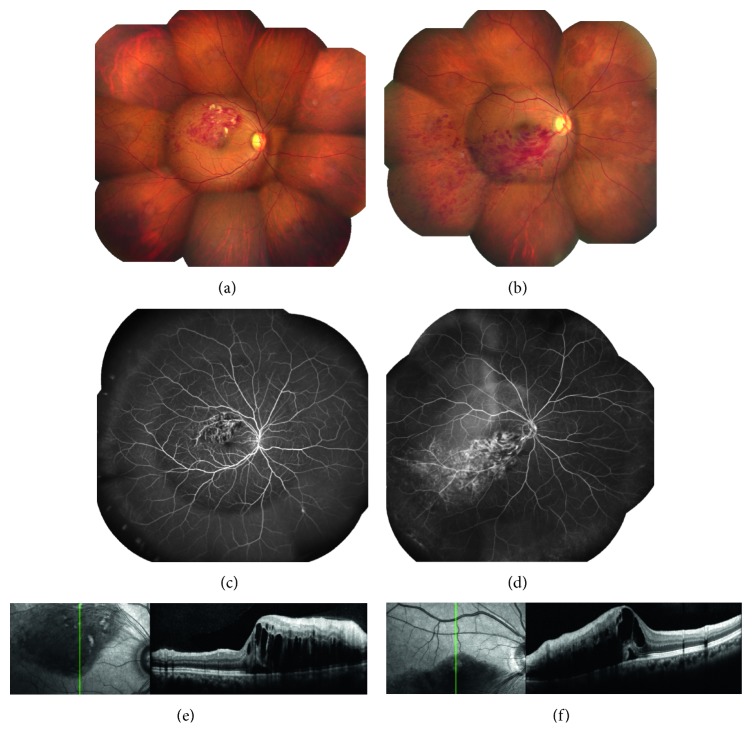
Color fundus photograph. (a) Macular branch retinal vein occlusion (BRVO). (b) Major BRVO. Fluorescein angiography. (c) Mild retinal hypoxia in macular BRVO. (d) Mild retinal hypoxia in major BRVO. Optical coherence tomography. (e) Macular edema and serous retinal detachment can occur in macular BRVO, even if retinal hypoxia is mild. (f) Macular edema and serous retinal detachment can also occur in major BRVO despite mild retinal hypoxia.

Because retinal arterioles and venules share a common adventitia at arteriovenous crossings, vein walls can be compressed by arteriosclerotic changes. Luminal narrowing of a venule leads to disturbance of laminar flow and endothelial damage due to shear stress, after which thrombus formation causes BRVO [3]. In the acute phase of BRVO, pressure increases in the affected capillaries and venules. Typically, brush-shaped hemorrhage is seen in the nerve fiber layer, and this finding is often associated with macular edema and serous retinal detachment. Cystoid spaces are formed as blood components leak into the retina and accumulate in the outer plexiform layer and inner nuclear layer [4]. For clinical management, BRVO is classified into major (first-order) and macular (second-order) subtypes based on the site of occlusion (Figures 1(a) and 1(b)) [5]. The clinical and angiographic findings of patients with macular BRVO are similar to those of patients with major BRVO in many respects. Severity of major BRVO depends on the affected vessel and a wide range of complications can occur. In contrast to major BRVO, patients with macular BRVO are unlikely to develop neovascularization because of the smaller area of the retina affected, but they frequently have macular edema and visual impairment [6]. Macular edema in patients with BRVO has been hypothesized to be caused by efflux of fluid from the affected vessels (Starling's law) [7, 8], due to breakdown of the blood-retinal barrier (BRB) as a result of damage to the tight junctions between capillary endothelial cells [9], vitreoretinal adhesion and traction on the macula [10], and secretion into the vitreous of vasopermeability factors such as VEGF [11] produced by retinal cells.

## 3. VEGF

When vitreous surgery was performed to treat macular edema in patients with BRVO, VEGF was detected in vitreous fluid samples obtained at operation. We have previously reported that the vitreous VEGF level was significantly higher in BRVO patients than in control patients with nonischemic diseases such as macular hole [12]. Although the most appropriate control would be BRVO patients without macular edema, we could not obtain data from such patients because it would not be ethical to sample vitreous fluid from BRVO patients who were not receiving clinically indicated vitreous surgery. Also, there are no papers reporting such data for the same reason. However, the available findings confirm that VEGF plays a role in the development of macular edema associated with BRVO, although cytokine levels in BRVO patients without macular edema should be measured in the future. Accordingly, treatment of macular edema in BRVO patients by inhibiting VEGF was attempted, resulting in dramatic improvement of edema.

VEGF is expressed by various cells in the retina after exposure to hypoxia, including retinal glial cells, retinal pigment epithelial cells, and vascular endothelial cells [13]. VEGF increases vascular permeability and promotes the proliferation of endothelial cells [14]. Vascular endothelial permeability is augmented because VEGF facilitates rearrangement of actin filaments in the cytoplasm and increases the phosphorylation of tight junction proteins such as zonula occludens-1 or occluding [15].

**Figure 2:**
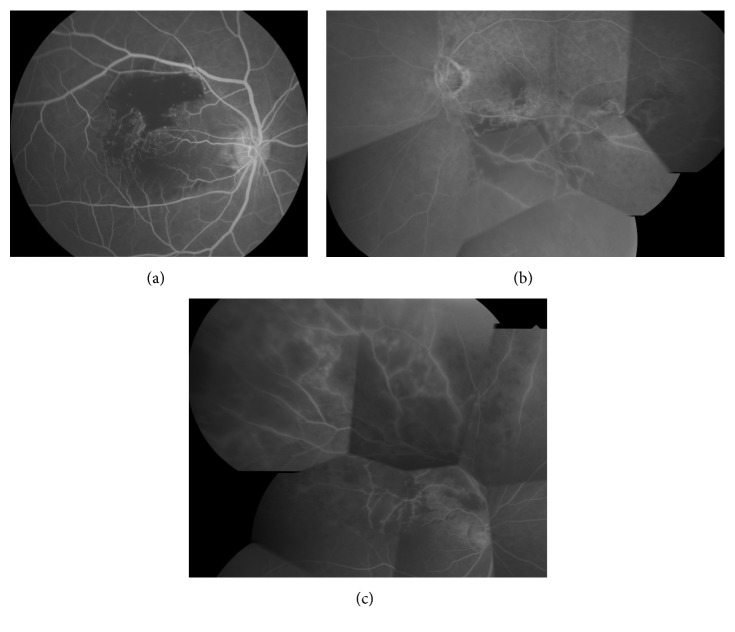
Vitreous fluid VEGF level and retinal nonperfusion in representative cases. Fluorescein angiograms. (a) Mild ischemia (15.6 pg/ml). The VEGF level is often 30–40 pg/ml in patients with mild nonperfusion. (b) Moderate ischemia (338 pg/ml). The VEGF level is often 300 pg/ml or higher when there is moderate nonperfusion. (c) Severe ischemia (2,570 pg/ml). The VEGF is often 2000–3000 pg/ml when nonperfusion is severe.

However, intraocular VEGF levels are not necessarily elevated in all BRVO patients [16], and VEGF levels actually show wide variation among patients. Accordingly, retinal nonperfusion (RNP) has attracted attention as the potential cause. When the ratio of the RNP area to the disc area was calculated as an indicator of the severity of retinal hypoxia, we found that the intraocular VEGF concentration was significantly correlated with the RNP area (Figure 2) [12]. The VEGF level also showed a significant correlation with the severity of macular edema [16]. These findings suggested that expression of VEGF initially increases after BRVO due to retinal hypoxia because of vascular occlusion, leading to disruption of the BRB and the development and progression of macular edema. Therefore, the pathogenesis of macular edema in BRVO patients seems to be closely associated with retinal hypoxia.

## 4. Inflammation and Other Cytokines

However, the pathological changes that occur in BRVO cannot be fully explained on the basis of retinal hypoxia. For example, macular edema can occur in BRVO, even if retinal hypoxia is mild (Figures 1(c)–1(f)). It was also reported that macular edema occurs in nonischemic BRVO patients with mild retinal hypoxia and that anti-VEGF therapy is more effective for nonischemic BRVO than ischemic BRVO [17]. Moreover, VEGF levels can be relatively high in BRVO patients with mild retinal hypoxia [12], suggesting that hypoxia is not the only factor involved in the pathogenesis of macular edema. What causes macular edema in patients with mild retinal hypoxia? The potential role of inflammation deserves consideration because the effectiveness of intravitreal steroid therapy has been demonstrated by a multicenter clinical trial in BRVO patients (Standard Care vs Corticosteroid for Retinal Vein Occlusion; SCORE) [18]. Furthermore, anti-VEGF therapy is ineffective for macular edema in some BRVO patients [19], suggesting that other factors/cytokines may also have a role in its development. Interestingly, it has been reported that the intraocular levels of monocyte chemoattractant protein-1 (MCP-1) (a chemokine), intercellular adhesion molecule-1 (ICAM-1) (an adhesion molecule), and interleukin-6 (IL-6) and IL-8 (inflammatory cytokines) are increased in BRVO patients with macular edema [20–24].

MCP-1 is a chemokine that promotes chemotaxis of monocytes. Its expression is increased by retinal hypoxia, arteriosclerosis, and oxidative stress [25–27]. MCP-1 promotes the phosphorylation of tight junction proteins, such as zonula occludens-1 or occludin [28, 29].

ICAM-1 is an adhesion molecule. ICAM-1 is expressed by normal retinal pigment epithelium cells, and its expression by various other cells in the retina and choroid (as well as leukocytes) has been demonstrated *in vivo* and *in vitro* [30]. In addition, the expression of ICAM-1 mRNA and protein is upregulated by retinal hypoxia [31, 32]. Upregulation of ICAM-1 expression induces leukostasis due to increased rolling and adhesion of leukocytes to vessel walls, leading to stagnation of blood flow [33]. After retinal vein occlusion *in vivo*, increased rolling of leukocytes and adhesion to vein walls have been confirmed to result in stagnant blood flow [34]. Thus, entrapment of leukocytes by adhesion to vascular endothelial cells could increase leakage from the retinal microvessels after BRVO.

IL-6 is a multifunctional cytokine that can increase endothelial permeability by inducing the formation of gap junctions between adjacent cells via rearrangement of actin filaments [35]. In cultured endothelial cells exposed to hypoxia, IL-6 mRNA expression increases in a time-dependent manner [36–38]. IL-8 is a potent chemoattractant and also activates neutrophils and T cells. Production of IL-8 is induced by exposure of vascular endothelial cells to hypoxia and oxidative stress [39–41], and IL-8 regulates endothelial permeability by downregulation of tight junctions [42, 43].

It seems likely that inflammation is involved in the pathogenesis of macular edema associated with BRVO because intraocular levels of these inflammatory factors and cytokines are increased in BRVO patients who develop macular edema.

The aqueous flare is an index of ocular inflammation that can be measured by using a laser flare meter, and it has been reported that aqueous flare values are significantly higher in BRVO patients than in control patients [44]. This is evidence of active inflammation in BRVO. In addition, the aqueous flare value shows a significant positive correlation with the intraocular levels of these inflammatory cytokines [44], so a higher flare value (which means greater inflammation) is associated with higher levels of inflammatory cytokines. In addition, major BRVO is associated with higher ocular fluid levels of inflammatory factors and higher aqueous flare values compared with macular BRVO [45–47], suggesting that inflammation is more severe in major BRVO, probably because of more extensive retinal hemorrhage. Moreover, not only the VEGF level but also the levels of the abovementioned factors/cytokines are significantly correlated with the severity of retinal hypoxia in BRVO [23, 24]. These findings are supported by the results of basic studies [28, 29, 35, 42, 43].

**Figure 3:**
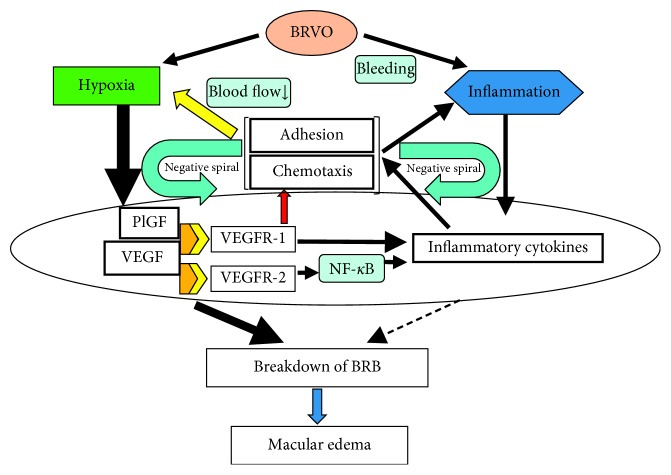
Pathogenesis of macular edema (hypothesis). BRVO not only causes retinal hypoxia but also produces inflammation secondary to retinal hemorrhage. As a result, expression of VEGF and inflammatory cytokines increases, resulting in disruption of the BRB with the development and progression of macular edema. Activation of VEGFR-1 by both VEGF and PlGF plays a role in recruitment of leukocytes and also upregulates expression of inflammatory cytokines. In addition, activation of VEGFR-2 by VEGF increases vascular permeability and enhances the expression of inflammatory cytokines such as MCP-1 and ICAM-1 via NF-*κ*B, leading to chemotaxis and adhesion of leukocytes to the vascular endothelium along with a decrease of blood flow velocity. Reduction of blood flow velocity creates a positive feedback loop, which further exacerbates retinal hypoxia. Increased leukocyte chemotaxis and adhesion also enhances inflammation, creating another positive feedback loop. According to this hypothesis, the pathological mechanism becomes more complicated and the influence of inflammation increases as BRVO becomes chronic.

Accordingly, it seems that BRVO causes both retinal hypoxia and inflammation. As a result, expression of VEGF and inflammatory cytokines is increased, resulting in disruption of the BRB with development and progression of macular edema (Figure 3). Among these factors/cytokines, it is considered that VEGF has a central role in the onset of macular edema because it strongly increases vascular permeability. Importantly, it is necessary for VEGF to bind to the VEGF receptor in order to activate signalling pathways that mediate its biological effects.

## 5. VEGF Receptors

VEGF signaling is initiated by binding to receptors, with two VEGF receptors (VEGFR-1 and VEGFR-2) being expressed in the retina. Binding of VEGF to either receptor activates autophosphorylation [48–50] and transphosphorylation induces a signaling cascade. We previously reported that aqueous humor levels of both VEGFR-1 and VEGFR-2 were significantly higher in BRVO patients than in controls [51], suggesting that these VEGF receptors could have an important role in the pathogenesis of macular edema associated with BRVO. VEGFR-1 is mainly expressed by monocytes/macrophages, and VEGFR-1 signaling plays a role in recruitment of leukocytes to sites of inflammation [52]. Placental growth factor (PlGF) is a member of the VEGF family [53, 54], and it also specifically binds to VEGFR-1, in addition to VEGF, stimulating tissue factor production and chemotaxis by monocytes/macrophages [55]. Binding of PlGF to VEGFR-1 increases the production of proinflammatory factors by cultured monocytes via a calcineurin-dependent pathway [56], suggesting that this molecule has a direct influence on the inflammatory response. Thus, activation of VEGFR-1 promotes inflammation [55, 57, 58]. On the other hand, VEGFR-2 is exclusively expressed by endothelial cells. Binding of VEGF to VEGFR-2 initiates signaling that not only increases vascular permeability but also upregulates the expression of inflammatory cytokines (such as MCP-1 and ICAM-1) via NF-*κ*B [59–61]. We previously suggested that inflammatory factors may be induced via NF-*κβ* in BRVO because we found that the vitreous fluid level of sVEGFR-2 was significantly correlated with the levels of several inflammatory factors, including sICAM-1 and MCP-1 [24]. These factors/cytokines induce chemotaxis of leukocytes and promote adhesion of inflammatory cells to the vascular endothelium, resulting in a further increase of vascular permeability. Thus, macular edema is promoted by VEGF binding to its receptors expressed by vascular endothelial cells, monocytes, and macrophages. In addition, there is clinical and experimental evidence that both sVEGFR-1 and sVEGFR-2 may influence vascular permeability during the inflammatory response since VEGF acts as a chemotactic factor for inflammatory cells via its receptors [62], suggesting that VEGF may promote inflammation as well as increasing vascular permeability.

## 6. Inflammation and Retinal Blood Flow Velocity

At this point, it is worth asking “what is inflammation?” Inflammation has been defined as “part of the complex biological response of body tissues to harmful stimuli.” When inflammation occurs, leukocytes accumulate at the site of the lesion and local blood flow velocity decreases. In fact, several previous studies have demonstrated that the retinal blood flow velocity is lower in BRVO patients than in persons with normal eyes [63–65]. We measured the retinal blood flow velocity by using scanning laser ophthalmoscopy with fluorescein angiography and detected moving particles in the capillary vessels [66]. These particles were considered to be leukocyte [67]. When we measured the retinal blood flow velocity in healthy controls, patients with hypertension, and BRVO patients, the reduction of blood flow velocity was correlated with the severity of BRVO [68, 69]. Thus, the finding of reduced blood flow implies that inflammation is active in BRVO. We hypothesized that injury to the vascular endothelium would increase the expression of adhesion factors such as ICAM-1, resulting in enhanced rolling and adhesion of leukocytes with reduction of the blood flow velocity in BRVO patients. In fact, when we evaluated the relation between retinal blood flow velocity and sICAM-1 in BRVO patients, we found that blood flow velocity showed a significant negative correlation with the vitreous fluid level of sICAM-1 [70], supporting our hypothesis (Figure 3).

## 7. Possible Mechanism of Macular Edema in BRVO

Summarizing the above (Figure 3), BRVO not only causes retinal hypoxia but also provokes inflammation due to retinal hemorrhage. As a result, intraocular expression of VEGF and various inflammatory factors/cytokines is increased, leading to BRB disruption with development and progression of macular edema. Activation of VEGFR-1 by VEGF and PlGF plays a role in recruitment of leukocytes, as well as upregulating the expression of inflammatory cytokines. In addition, activation of VEGFR-2 by VEGF not only influences vascular permeability but also increases the expression of inflammatory cytokines such as MCP-1 and ICAM-1 via NF-*κ*B, leading to chemotaxis of leukocytes and adhesion of inflammatory cells to the vascular endothelium. These changes reduce local blood flow velocity and create a positive feedback loop [71], which further promotes retinal hypoxia. Increased leukocyte chemotaxis and adhesion also enhances inflammation, creating another positive feedback loop. One of the mechanisms for progression from the nonischemic type to the ischemic type may be a positive cytokine feedback loop due to inflammation, although it was reported that transformation from the nonischemic to the ischemic type takes some time [72]. That is, inflammatory cytokines such as MCP-1 and ICAM-1 are increased by this positive feedback loop, leading to leukocyte abnormalities and a reduction of retinal blood flow velocity with transformation to the ischemic type. According to this hypothesis (Figure 3), the pathological mechanism becomes more complicated in chronic BRVO as the influence of inflammation increases. For example, when macular edema becomes chronic, the positive feedback loop leads to a gradual increase of VEGF and inflammatory cytokines, resulting in refractory edema. Thus, the positive cytokine feedback loop seems to be one of the factors contributing to chronic macular edema. Since VEGF may be a critical cytokine in this positive feedback mechanism, it should be important to inhibit VEGF as soon as possible in order to block the establishment of positive feedback.

In fact, the mean change of BCVA from the baseline after 12 months was larger in patients with a shorter duration of BRVO before the initiation of treatment with ranibizumab [73], suggesting that early anti-VEGF therapy blocks the positive feedback loop. That is, VEGF is inhibited by anti-VEGF agents, leading to decrease of leukocyte rolling and adhesion (leukostasis) that prevents the positive feedback loop from being established. We have previously reported that the number of doses of anti-VEGF therapy is significantly correlated with the baseline aqueous levels of inflammatory cytokines (sICAM-1, IL-6, and IL-8) [74]. Therefore, early initiation of anti-VEGF therapy should be considered to block positive feedback before macular edema becomes chronic. Anti-VEGF therapy inhibits the positive feedback loop because the levels of downstream inflammatory cytokines are reduced in addition to VEGF itself. Monthly anti-VEGF therapy results in a significant decrease of downstream inflammatory cytokines (such as MCP-1 and IL-8), as well as VEGF [75], leading to improvement of retinal hypoxia and inflammation. In a study of monthly ranibizumab treatment for RVO, there was no increase of retinal hypoxia in the active drug group, but patients with RNP increased in the placebo group and there was a significant difference after 6 months. After 6 months, patients in the placebo group also received intravitreal ranibizumab and the percentage of patients without RNP increased until there was no longer any difference between the two groups [76]. Thus, aggressive VEGF blockade prevents the progression of RNP and promotes reperfusion by controlling the positive feedback loop [71]. In addition, the aqueous flare value showed a significant decrease at both 1 and 6 months after anti-VEGF therapy [77], suggesting that this therapy inhibited inflammation. Accordingly, anti-VEGF therapy seems to improve retinal hypoxia and inflammation in BRVO patients by inhibiting both VEGF and inflammatory cytokines to suppress leukocyte chemotaxis and adhesion in the retinal vessels. For example, it was reported that treatment of macular BRVO requires fewer doses of anti-VEGF therapy than major BRVO [45, 47]. The positive feedback loop may not be fully established in macular BRVO due to less extensive retinal hypoxia and inflammation. Therefore, macular BRVO may be less likely to progress.

However, the BRAVO [78] and HORIZON [79] studies showed that the mean change of BCVA from the baseline after 12 months was larger in BRVO patents receiving anti-VEGF therapy than in the placebo group, although there was no difference between the two groups after 24 months. From these findings, it is still unclear whether early anti-VEGF treatment is necessary, and early initiation of anti-VEGF therapy remains controversial. Moreover, it is well known that macular edema resolves spontaneously without treatment in some BRVO patients [80]. One possible reason is that the positive cytokine feedback loop may not function due to differences in the activity of signaling pathways [81], resulting in spontaneous resolution of macular edema.

## 8. Conclusion

We reviewed the pathogenesis of BRVO from the perspective of cytokines. In addition to VEGF, various inflammatory cytokines promote the development of inflammation and retinal hypoxia in the pathogenesis of BRVO, and the underlying mechanism is complex. Generally, anti-VEGF agents are effective if used early after the onset of BRVO because VEGF is produced in response to retinal hypoxia accompanying vascular obstruction and is the predominant actor in the early period. However, as shown in Figure 3, a positive feedback loop forms over time. As a result, expression of inflammatory cytokines increases and inflammation becomes more severe, with the disease becoming complicated and resistant to anti-VEGF therapy. This cytokine hypothesis regarding the pathogenesis of BRVO seems to be plausible considering the available data and could also be useful for developing new therapeutic strategies.
